# Dental Jottings

**Published:** 1873-03

**Authors:** H. L. Sage


### Dental Register.

Vol. XXVII.] MARCH, 1873. No. 3.]

### COMMUNICATIONS.

### Dental Jottings.

BY H. L. SAGE, D. D. S.

*Abscess of the Cheek Produced by the Eruption of an Inferior Dens Sapientiæ*

Mr. D—— (teacher) presented June 24th, 1872, with jaws very nearly closed, barely admitting the extreme point of the forefinger.

Right cheek much swollen, and very hard from the infiltration of lymph—the portion in front of the parotid gland, and about opposite the point of antagonism of the second and third molars, being involved.

The anterior third of the grinding surface of the right inferior dens sapientiæ, was just visible, the remaining portion being overlapped with gum, and the crown presenting towards and against the posterior proximal surface of the second molar.

The left inferior wisdom tooth presented about the same progress in eruption, though, perhaps, a trifle more advanced, but has not produced marked irritation.

The remaining teeth are regular, firm, sound, dense, and very close together.

Pressure on the swelling from the inside of the cheek, which could be made with a thin, flat instrument, (for instance, the handle of a pearl-handle mouth glass) caused a considerable discharge of pus, from the gums, covering and overlapping the buccal and grinding surfaces of the tooth.

The history of the case is as follows: In January, 1868, while the patient was in Iowa, the trouble commenced by inflammation and soreness of the part, with difficulty of deglutition, which resulted in nearly closing the mouth, leaving, when opened as far as possible, a space between the cutting edges of the incisors, of only an eight of an inch—the inflammation lasting several weeks. In the summer of 1871, the same trouble occurred, but with less severity, and was of shorter duration. In March 1872, a slight attack of the inflammation recurred. Before calling on me in June, 1872, the time of the fourth attack, the patient consulted a physician, who affirmed that a tooth was not the cause of the swelling, though he was unable to make a definite diagnosis of the case. Subsequently he thought it might proceed from an "ulcerated tooth." Another physician being consulted, could not locate the cause of the swelling.

My diagnosis was, that this chronic case of retarded or resisted dentition, caused by the want of room between the ramus of the jaw and the second molar, produced irritation and inflammation of the integuments about the tooth, which involving the looser, or more vascular cellular tissue of the cheek, caused the greatest tumefaction therein, because of its more easy and rapid infiltration with lymph, than the contiguous and less vascular portions more immediately affected.

The great resistance offered to the eruption of the tooth, had, at previous times, caused inflammation only in the muscles or tissues, which are usually involved in such cases, and which terminated in resolution, but had, during the last attack, passed on to suppuration, the pus, perhaps, burrowing along under the fascia, until it reached the vascular tissues of the cheek, therein locating and circumscribing the swelling—the tense and indurated state of the masseter, and other muscles and integuments, also preventing the opening of the mouth. That the swelling, though situated quite a distance from the exciting cause, was the result of irritation, produced by the resisted eruption of the dens sapientiæ, would seem apparent from the fact that pressure upon it, caused a discharge of pus between the gum and the tooth, about the buccal portion. Or the inflammation may have been sympathetic, i. e., the abscess of the cheek, disconnected from the discharge about the tooth, may have been dependent upon the same exciting cause.

When my attention was first called to this case, it seemed desirable to check the irritation by the extraction of the dens sapientiæ, if not too difficult and hazardous an operation, or, if it seemed necessary or desirable in preference, the removal of the second molar. But in the conditions then presented, this could not easily be effected, neither did the operation seem immediately imperative.

So, with difficulty, an injection was made, with a hypodermic syringe, of the elixir of vitriol, under the gum in the buccal region, from whence it was at that time discharging, and an application to the swelling on the inside, of the tinct. iodine and aconite, equal parts. This caused considerable pain for two hours, but on the following day the swelling was more circumscribed, with less soreness of the parts, and the patient was able to open his mouth a trifle farther—and as he expressed it, " he had not felt so well for a fortnight."

The discharge about the neck of the tooth had also nearly ceased. Treatment was continued, and gradual improvement was being made, so that the patient was able to insert his forefinger between the cutting edges of the incisors, to the middle joint, though the forceps could not be entered sufficiently to clasp even the second molar. The swelling, at this stage of the treatment, was yet indurated, with no fluctuation on pressure with the finger, when the patient left town on a vacation, being absent over a month, and on his return consulted a third physician, who finding the cheek had suppurated, lanced it on the inside, just forward of the parotid gland. This was followed by a copious discharge of pus, but afforded only partial relief, for when I afterwards met the patient on the street, the abscess was discharging on the outside, just back of the angle of the mouth, and so continued to do for several months, when, November 22, 1872, he informed me that the fistula had, within a week, entirely closed up.

The swelling when last under my observation and treatment, (June 29, 1872) was so indurated, that lancing did not, at that time, appear to be indicated. During the absence of the patient, the softening of the abscess, by the secretion of pus, rendered this advisable, but, as the sequel proved, did not prevent a final discharge from the outside—though, had the treatment been followed up, this might have been prevented.

So that what appeared to be an unnecessary deformity, was, perhaps, chargeable to the negligence of the sufferer, who discontinued his visits as soon as the incision was made by the physician. As the fistula discharged only pus, the parotid gland, if in a measure involved, was not sufficiently so, to interfere with its functions, particularly as there was no closure of the duct of steno.

*Aveolar Abscess Caused by Salivary Calculus.*

A gentleman, probably between thirty and forty years of age, recently called for advice in regard to the right, inforior, sixth-year molar—a prominent and circumscribed swelling, having appeared on the lingual surface of the gum, about mid-way of the bifurcation of the roots.

On lancing it, a considerable quantity of pus was discharged. A diagnosis revealed the presence of tartar on the cervical portion of the tooth, above the swelling, with recession of the margin of the gum, and a loss of connection for a distance corresponding to the length of the roots, with the absorption of the lingual portion of the process. The presence of tartar, could not be detected much below the margin of the receded gum, but may it not have been dissolved, and removed by the same process as are, sometimes, the apexes of roots in alveolar abscess?

With the exception of two small cavities on the buccal and grinding surfaces, which had been filled with gold, the tooth was sound, and of very fine formation and texture, as were all of the others, exhibiting, by their positions, regular and symmetrical arches.

The gums and alveoli over the buccal portion of the roots were healthy, and the tooth, by its appearance, gave no signs of impaired vitality, being normal in color—neither was there any soreness or looseness about it, but the reverse was true. The indications were, that the periosteum lining, and covering the buccal, anterior, and posterior portions of the roots and sockets, still maintained its vitality.

After the instrumental removal of the calcareous deposit, an injection of the elixir of vitriol was made to the effected parts, for the purpose of obtaining the benefit of its astringent, caustic or escharotic, and stimulant properties, and for the effect it might have in dissolving any particles of tartar that might remain. Though this was followed by a partial improvement, there does not appear to be any probability in this case, of restoring the *fnll* integrity of the parts, even if control of the patient is had, which is not likely to be obtained. Perhaps, with systematic and thorough treatment this might be accomplished, but this would necessitate a restoration of alveolus, and of periosteum, of which the lingual surfaces of the roots are denuded. This case is cited, because the production of alveolar abscess in this *location*, by a deposit of salivary calculus, is rare.

*Encystment of a Superior Cuspidatus.*

A young woman, aged twenty-five years, had her upper teeth or roots extracted, May 31, 1869, preparatory to the insertion of an artificial denture.

At a subsequent examination of the mouth, it was discovered that absorption of the alveolus had not taken place in the region of the socket of the left cuspidatus—a prominence of the process (as is observed just previous to the eruption of these teeth) being quite apparent, for which reason the presence of a tooth was suspected, when, on cutting through the bone on the labial surface, a perfect canine tooth was found imbedded therein—presenting no deviation from the ordinary position of a tooth in the normal process of eruption. It was removed without difficulty.

### *Case of Retarded Dentition.*

A young woman of exceedingly nervous temperament, recently called (March 30, 1872) for the extraction of the left second superior temporary molar, to make room for the second bicuspid, which was just appearing, as well as to relieve her of much suffering, of which she complained. The two buccal roots were absorbed, but the palatine was entire, and brought with it, on extraction, the sack of the abscess.

The left superior cuspidatus was also just cutting irregularly, and back of the lateral incisor of the same side. The other teeth, with the exception of the first left superior bicuspid, which was absent, were fully erupted and regular.

As the patient was nineteen years of age, or so stated, though she appeared to be older, this may be cited as a case of retarded dentition, the canine being severe, and the second bicuspid eight years behind the usual average time of appearance.

In the case of a married lady, aged thirty years, with children, and judging from appearance in good health, the right and left second inferior temporary molars were yet firm and sound.

While we often observe cases, where some of the temporary teeth, more generally the canines, remain to adult age, it is not common for the corresponding permanent teeth to make their appearance if delayed far beyond the average period, for which reason we hesitate to extract such temporary teeth, unless an urgent necessity exists for so doing.

